# Social Psychology: Conspiratorial contradictions

**DOI:** 10.1038/s44271-023-00010-3

**Published:** 2023-08-08

**Authors:** Jennifer A. Bellingtier

**Affiliations:** Communications Psychology, https://www.nature.com/commspsychol

**Keywords:** Human behaviour

## Abstract

Does endorsing one conspiratorial belief make you more likely to endorse a second, incompatible, conspiracy? A recent study in *Psychological Science* suggests that past work identifying this pattern may actually be driven by those who reject both.

**Figure:**
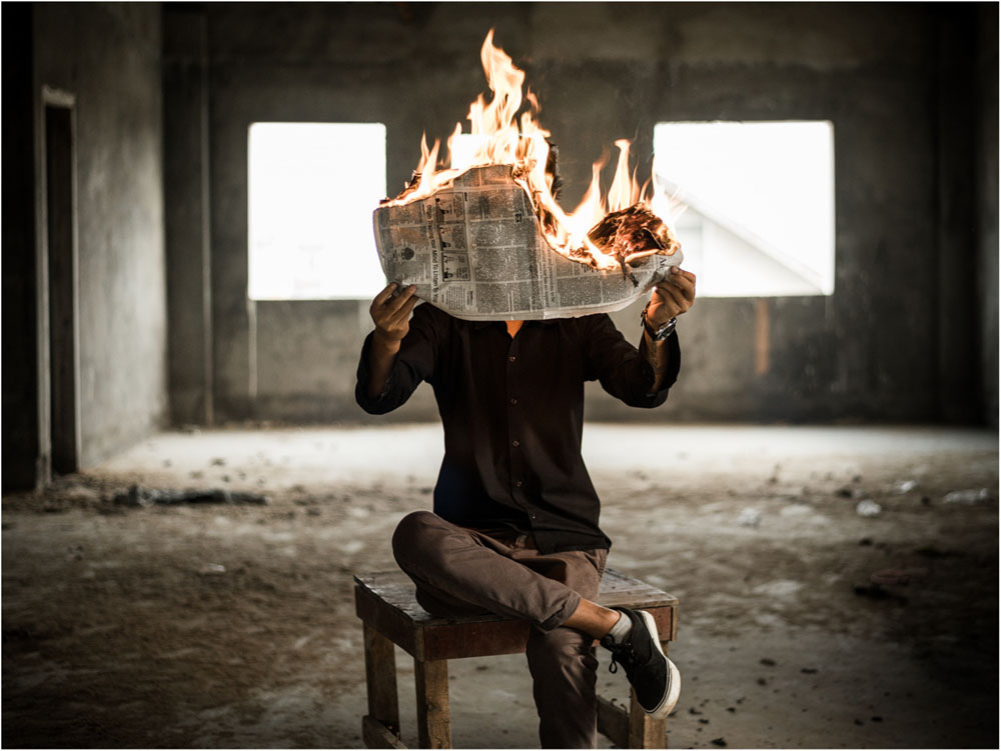
Nijwam Swargiary on Unsplash

Contradictory conspiracy theories abound for many topics, for example, the belief that Hitler escaped to Argentina is incompatible with the theory that he was killed by the Russians. Past research has consistently found a positive correlation between these beliefs. This has been interpreted as evidence that those who endorse one conspiracy theory are more likely to endorse another, even when they are in direct conflict with each other.

Jan-Willem van Prooijen and colleagues from Vrije Universiteit Amsterdam, questioned if the positive correlation between contradictory conspiracy theories was driven by these individuals who believed Hitler was both dead and alive^1^. In a series of four studies, they instead demonstrated that the correlation was driven by individuals who rejected both conspiracy beliefs. By asking participants if they believed the official version of events, they were able to calculate correlations separately for each group. Consistent with past work, the overall correlation was positive, as was the correlation for those who accepted the official version of events. However, the correlation for those who rejected the official version of events was mostly negative or non-significant. Most people do not believe Hitler is both dead and alive.

These findings challenge the traditional view of a “conspiratorial mindset” and raise the question of positive correlations between non-incompatible conspiracy beliefs also being due largely to individuals who disbelieve both.
